# Enhancing thermal performance of phase change materials using conductive rods with length dependent melting dynamics

**DOI:** 10.1038/s41598-025-17040-y

**Published:** 2025-08-26

**Authors:** Abbas Fadhil Khalaf, Farhan Lafta Rashid, Mudhar A. Al-Obaidi, Hayder I. Mohammed, Arman Ameen, Ephraim Bonah Agyekum

**Affiliations:** 1 Department of Petroleum Engineering, Engineering College, University of Kerbala, Karbala, 56001 Iraq; 2 Technical Instructor Training Institute, Middle Technical University, Baghdad, 10074 Iraq; 3 Department of Cooling and Air Conditioning Engineering, Imam Ja’afar Al-Sadiq University, Baghdad, 10011 Iraq; 4 Department of Building Engineering, Energy Systems and Sustainability Science, University of Gävle, 801 76 Gävle, Sweden; 5 Department of Nuclear and Renewable Energy, Ural Federal University Named After the First President of Russia, Boris Yeltsin, 19 Mira Street, Ekaterinburg, Russia 620002; 6 Western Caspian University, 31, Istiglaliyyat Street, AZ1001 Baku, Azerbaijan; 7 Istanbul Okan University, Tuzla Campus, 34959 Tuzla, Istanbul, Turkey

**Keywords:** Phase change materials (PCMs), Heat transfer enhancement, Thermal energy storage, Natural convection, Copper rod, Enthalpy-porosity model, Energy science and technology, Engineering, Materials science

## Abstract

Phase change materials (PCMs) suffer from slow melting rates due to their low thermal conductivity, limiting their efficiency in thermal energy storage systems. This study numerically investigates the novel use of copper rods as conductive enhancers to accelerate PCM melting in a horizontally placed hemispherical cell. Using the ANSYS/FLUENT 16 with an enthalpy-porosity model, the impact of rod integration is examined to determine the optimal rod configuration for maximising heat transfer while minimising melting time. The results indicate that copper rods dramatically improved melting performance: a 20 mm rod can reduce total melting time by 70% (from 300 to 90 min), while 10 mm and 15 mm rods achieve reductions of 40% (to 180 min) and 50% (to 150 min), respectively. Clearly, the 20 mm rod enables 70% liquid fraction in 30 min, showing a melting speed four times faster than the no-rod case. Nonlinear scaling reveals diminishing returns beyond 15 mm, suggesting a cost-performance trade-off at this length. The 15 mm rod emerged as a practical balance between attaining 85% of maximum gain with a 50% reduction in melting time while utilising 25% less copper than 20 mm rod. Accordingly, this research provides critical insights for designing high-efficiency thermal storage systems, offering a roadmap to optimise conductive enhancements for real-world applications. By bridging the gap between material properties and system-level performance, the findings advance the deployment of PCMs in renewable energy and waste heat recovery systems.

## Introduction

Energy plays a pivotal role in modern society, driving technological advancement and economic development while directly impacting daily life. However, conventional energy sources face two critical challenges: environmental degradation caused by fossil fuel use and the finite nature of non-renewable resources. These limitations have spurred global efforts to transition toward renewable energy alternatives such as solar and wind power^1,2^. While these renewable sources offer sustainable potential, their intermittent availability, due to factors such as the absence of sunlight at night or inconsistent wind patterns, remains a key barrier to a reliable energy supply^3–5^. Thermal energy storage (TES) using phase change materials (PCMs) presents a viable solution to bridge this intermittency gap^6^. PCMs can store and release large amounts of energy during phase transitions, making them ideal for mitigating supply–demand mismatches in renewable energy systems. Their applications range from solar energy storage to waste heat recovery in industrial processes. However, a significant limitation of PCMs is their inherently low thermal conductivity, which slows heat transfer and reduces overall system efficiency.

Extensive research has examined the relationship between system geometry and thermal performance in PCM-based TES systems. Studies have shown that container shape significantly influences heat transfer dynamics during both melting and solidification. Among various thermal enhancement strategies, copper rods have proven particularly effective due to their high thermal conductivity (401 W/mK), which facilitates efficient heat transfer pathways within PCM storage systems^7^. Numerical modelling has been instrumental in optimising these systems, with computational predictions often correlating strongly with experimental results. The integration of simulation and physical testing enables more efficient and accurate system design^7^. Research conducted in rectangular enclosures has revealed two key phenomena: (1) melting rates exhibit an exponential dependence on temperature differentials, and (2) the contribution of sensible heat becomes increasingly significant at higher operating temperatures^8^. These findings have been further validated by studies showing that elevated inlet air temperatures accelerate phase change processes and substantially enhance both sensible and latent heat storage capacities^9,10^. Further studies elucidated that melting progresses more rapidly as heat input increases^11,12^. Hlimi et al.^13^ conducted a numerical simulation to analyse thermal energy storage in a cylindrical geometry filled with PCM. Due to the elevated temperatures at the core of the PCM cylinder, the first row of cylinders in the column began to melt earlier than the surrounding cylinders. Experimental studies using horizontally oriented cylindrical capsules revealed that conduction dominates the initial stages of PCM melting^13^. As the process advances, natural convection becomes the governing mechanism, significantly influencing the overall melting duration. Further research was presented by Bechiri and Mansouri^14^ to investigate partial melting in vertically oriented cylindrical tubes. This demonstrated that the melting behaviour is affected by several factors, including pipe diameter, external wall temperature, thermos-physical properties of the PCM, and the thickness of the tube shell^14^. Additional experiments were carried out using spherical containers to study PCM melting. These studies indicated that increasing the heater lead wattage accelerates the melting process^15,16^. In a related investigation focusing on spherical PCM cells, it was observed that increasing the cell lead diameter results in a reduction in melting rate^17^.

Recent studies have significantly enhanced our understanding of PCM behaviour in various geometric configurations through combined numerical and experimental methodologies. Numerical simulations of spherical PCM cells have revealed distinct convection patterns, with the upper hemisphere exhibiting 25–40% stronger natural convection effects compared to the lower hemisphere. This asymmetry arises from buoyancy-driven flow and has important implications for the design of spherical thermal storage systems, suggesting that asymmetric heating strategies or internal baffles may improve melt uniformity^18^. Shell-and-tube configurations have been widely investigated for solar thermal applications. Experimental data indicated that natural convection is the predominant heat transfer mechanism, contributing 60–75% of the total energy transfer during melting^19,20^. The buoyancy effect leads to a characteristic top-down melting pattern, reducing the total phase change duration by up to 30% compared to conduction-dominated systems. Multi-tube arrangements offer promising improvements; increasing the number of tubes from 1 to 4 can enhance melting rates by 50–65%, while a 10 °C increase in operating temperature typically accelerates melting by 20–25%^21^. These systems benefit from optimised tube placement that balances heat transfer area with available PCM volume^22,23^. Nanoparticle-enhanced PCMs have emerged as a breakthrough technology, with experimental studies reporting 40–70% improvements in thermal conductivity by adding 1–5% volume fraction nanoparticles (e.g., CuO, Al_2_O_3_)^24,25^. Nanofluid-PCM composites demonstrate even greater potential, achieving up to 80% reduction in melting time through synergistic effects of improved conductivity (120–150% increase) and modified nucleation characteristics^26–30^. These advanced materials showed promise for high-power applications where rapid charge/discharge cycles are critical, though long-term stability studies indicate some degradation after 500–1000 thermal cycles. Despite the widespread use of PCMs in thermal energy storage, their low thermal conductivity remains a critical bottleneck, leading to inefficient melting/solidification rates and limiting practical applications^31,32^.

While previous studies have explored conductive additives like nanoparticles or fins, the systematic optimisation of embedded copper rods (particularly their length-dependent effects in hemispherical enclosures) remains underexplored. Thus, this research intends to improve the thermal performance of PCMs by integrating conductive copper rods, thus hastening melting rates and enhancing efficiency in thermal energy storage systems. In this regard, it should be noted that copper has a high score of thermal conductivity (401 W/mK), which is important to optimise the heat transfer efficiency of PCM systems. To systematically conduct this research, the used methodology comprises a numerical analysis using ANSYS/FLUENT 16 software, which uses an enthalpy-porosity model to accomplish detailed simulations of heat transfer dynamics. The experimental setup comprises exploring the melting behaviour within a horizontally oriented hemispherical cell, integrating copper rods of variable lengths (10 mm, 15 mm, and 20 mm) into the PCM. Total melting time and liquid fraction are associated as the performance metrics to be assessed at specific intervals to count the effect of rod integration. A comparative analysis is achieved against a no-rod baseline to evaluate the efficacy of the copper rods in improving heat transfer and mitigating melting time. Also, nonlinear scaling analysis inspects the relationship between rod length and melting efficacy, determining optimal configurations for practical deploying. Thus, the used detailed methodology permits for an intensive assessment of both thermal dynamics and material efficacy in PCM applications. Expectedly, this research would offer actionable insights for renewable energy storage and waste heat recovery applications.

## Numerical procedure

### Physics models

**Fig. 1:**
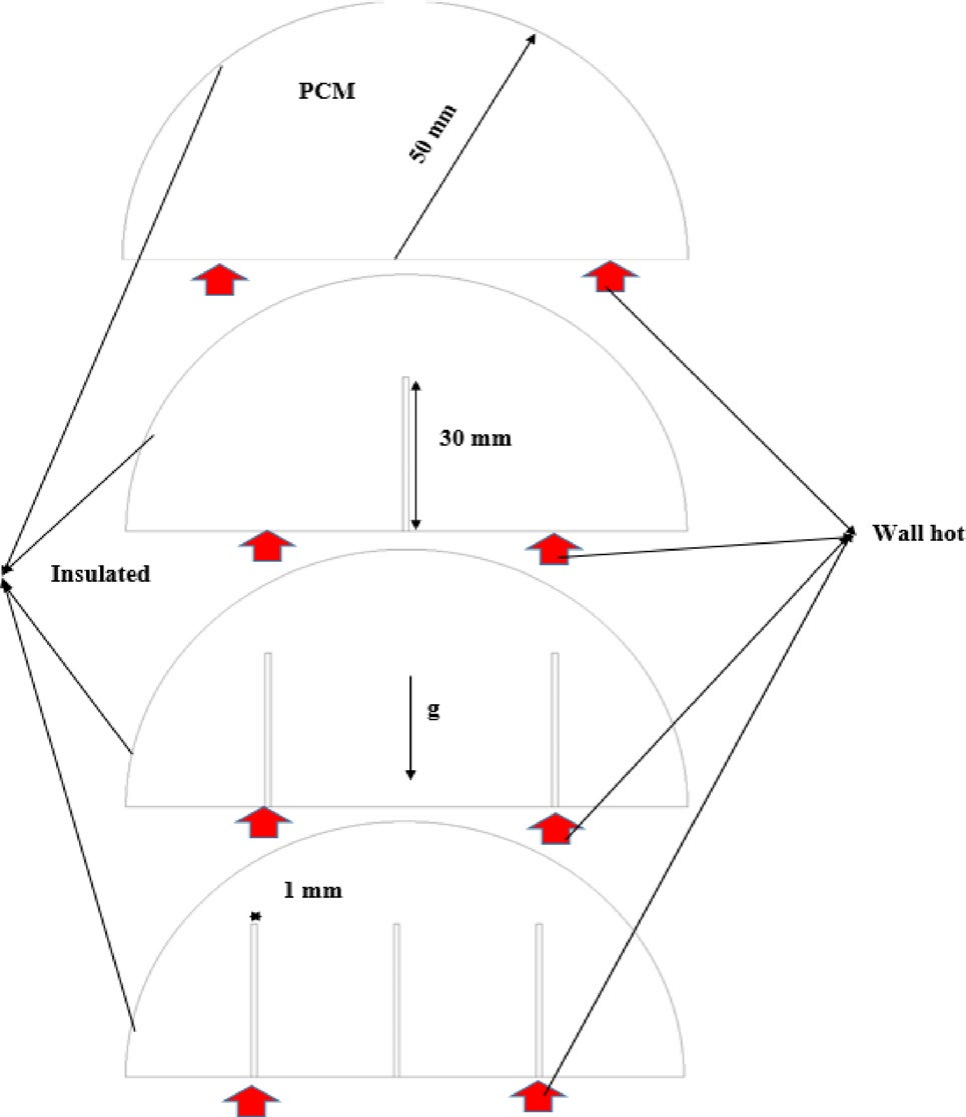
Configuration of physical model.

The studied cylindrical cell with a diameter of 50 mm is filled with phase-changing materials, and a copper rod with diameters (10, 15, and 20 mm) is used, as shown in Fig. 1.

### Computational procedure

The ability to predict the detailed behaviour of the melting process within the half-cylindrical cell is carried out by using numerical simulations. The flow is modelled as laminar, unsteady, incompressible, and two-dimensional. It is assumed that both the liquid and solid phases are homogeneous, isotropic, and maintain thermal equilibrium at their interface during the melting process. To simulate the phase change, the enthalpy–porosity method is employed to model the PCM melting zone^33,34^. The solid–liquid interface is characterized by continuous movement, nonlinear behaviour, and time dependency, features intrinsic to the melting dynamics of PCMs, which are treated as a complex, multi-physics process. The PCM melting process is governed by the coupled conservation equations of mass, momentum, and energy, which is shown in Eqs. 1–3^35^:

1 $$\frac{\partial \rho }{\partial t}+ \nabla \cdot \left(\rho V\right)=0$$

2 $$\frac{\partial (\rho v)}{\partial \text{t}}+ \nabla \cdot \left(\rho V\right)= - \nabla P+ \mu {\nabla }^{2}V+ \rho g\beta (T-{T}_{ref})+S$$

3 $$\frac{\partial }{\partial t}\left(\rho H\right)+ \nabla \cdot \left(\rho VH\right)= \nabla \cdot \left(K\nabla T\right)$$

The specific enthalpy *H* is the sum of the sensible enthalpy (h) and the latent heat (Δ*H*) and is calculated by using Eqs. 4–7 as shown below^36^:

4 $$H = h + \Delta {\text{H}}$$

5 $$h={h}_{ref}+\underset{{T}_{ref}}{\overset{T}{\int }}{C}_{p} dT$$

6 $$\Delta H=\beta {L}_{f}$$

The meaning of the latent heat ability varies between zero (for a solid) and one (for a liquid), and the liquid fraction (*β*) can be written as:

7 $$\beta = \left\{ {\begin{array}{*{20}c} {0\,solidus} & {if\,T < T_{s} } \\ {1\,liquidus} & {if\,T > T_{l} } \\ {\frac{{T - T_{s} }}{{T_{l} - T_{s} }}} & {if\,T_{s} \le T \le T_{l} } \\ \end{array} } \right\}$$

In this aspect, the melting time can be defined as the duration required to achieve complete melting (β = 1) of the PCM, as this metric can provide a clear and consistent benchmark for comparing the performance of different rod configurations.

The source term *S* in the momentum equation represents the Darcy damping term, which is incorporated to account for the effect of phase change on convective flow. This term is calculated using Eq. 8, as shown below:

8 $$S=\frac{{C(1-\beta )}^{2}}{{\beta }^{3}}V$$

*C* is the mushy zone constant, which reflects the morphology of the melting front. This constant plays a critical role in controlling the flow resistance in the mushy region and typically ranges between 10^4^ and 10^7^. In the present study, *C* is assumed to be constant and is set to 10^5^.

### Boundary conditions

The cylindrical cell under investigation is thermally insulated on three sides, with heat transfer occurring through the remaining side. The initial temperature parameters were also set at 298.15 K (25 °C), the set temperature is 13–17 K below the melting range (311.15–315.15 K), thus a full solid state was obtained at the beginning of the simulation. The addition of this conservative margin was made to wipe out any pre-existing liquid fraction ensuring that the dynamics of melting were exclusively dissolved by the prescribed thermal boundary conditions. In the simulations, constant temperature condition of 60 °C was applied to the heated wall of the hemispherical cell and all other three walls of the cell are thermally insulated to isolate the effect of heat transfer details of the copper rods. This experimental arrangement was adopted so as to be able to generate scenarios between reality of the thermal energy storage: localized heating is conventional. Properties of the paraffin wax (RT42) PCM such as the melting range (311.15–315.15 K) were strictly characterized (Table 1), and the enthalpy-porosity model was used to take into consideration the dynamics of the phase-change process. Specifically, the thermal properties of paraffin wax are listed in Table 1. Such a plan is consistent with previous work on PCM (e.g., Dhaidan and Khalaf^37^ and Sharma et al.^6^) and guarantees replicability, since it does not introduce ambiguities in phase distribution at an initial phase, but includes a clear baseline, against which the conductive enhancement effects of the rods can be measured.

**Table 1:** Thermal properties of the Paraffin (RT42) ^6^.

Properties	RT42
Density, *ρ* (kg/m^3^)	760
Specific heat capacity, *C*_*p*_ (J/kgK)	2000
Thermal conductivity, *k* (W/mK)	0.2
Dynamic viscosity, *μ* (kg/ms)	0.02351
Thermal expansion rate, *α* (1/K)	0.0005
Latent heat,* L* (J/kg)	165,000
Melting temperature, *T*_*m*_ (K)	311.15–315.15

### Assumptions

**Fig. 2:**
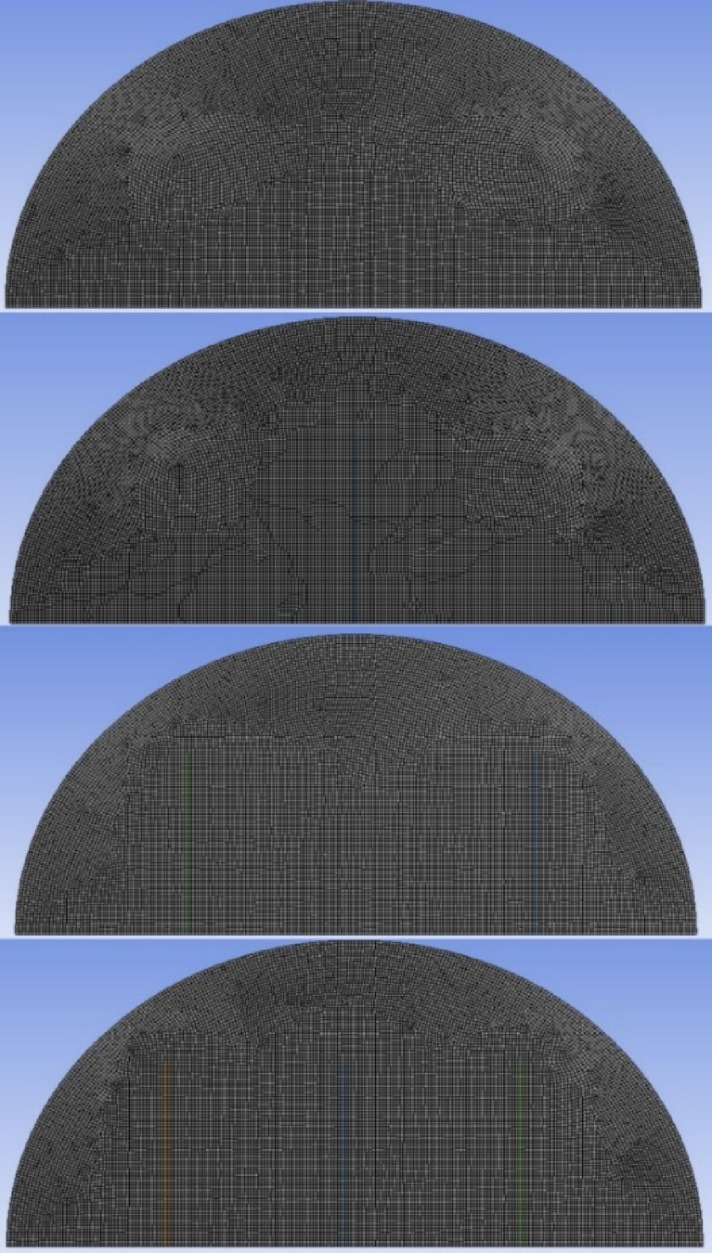
Configurations of mesh model.

In formulating the mathematical model to describe the melting process within a rectangular cell, several assumptions are considered. The melting is modelled in two dimensions. The flow is assumed to be unsteady, laminar, and incompressible. Viscous dissipation is neglected, and the effects of volume change due to the solid–liquid phase transition are also not considered. It is further assumed that there is no heat gain or loss from the surrounding environment. The thermal properties of the PCM are considered constant in both the solid and liquid phases throughout the simulation. Figure 2 shows the mesh distribution used in the computational model.

The model also assumed ideal thermal contact between the copper rods used and PCM because the thermal conductivity of copper is high (401 W/m K) and the interfacial resistance was non-existent in comparable PCM-conductive enhancer systems when operated at steady-state conditions. This assumption agrees with previous research^35,37^ in which estimate results during the experiment revealed that little departure occurred between calculated (through simulation) and measured result when interface impacts were insubstantial.

### Grid independence and the code validation tests

**Fig. 3:**
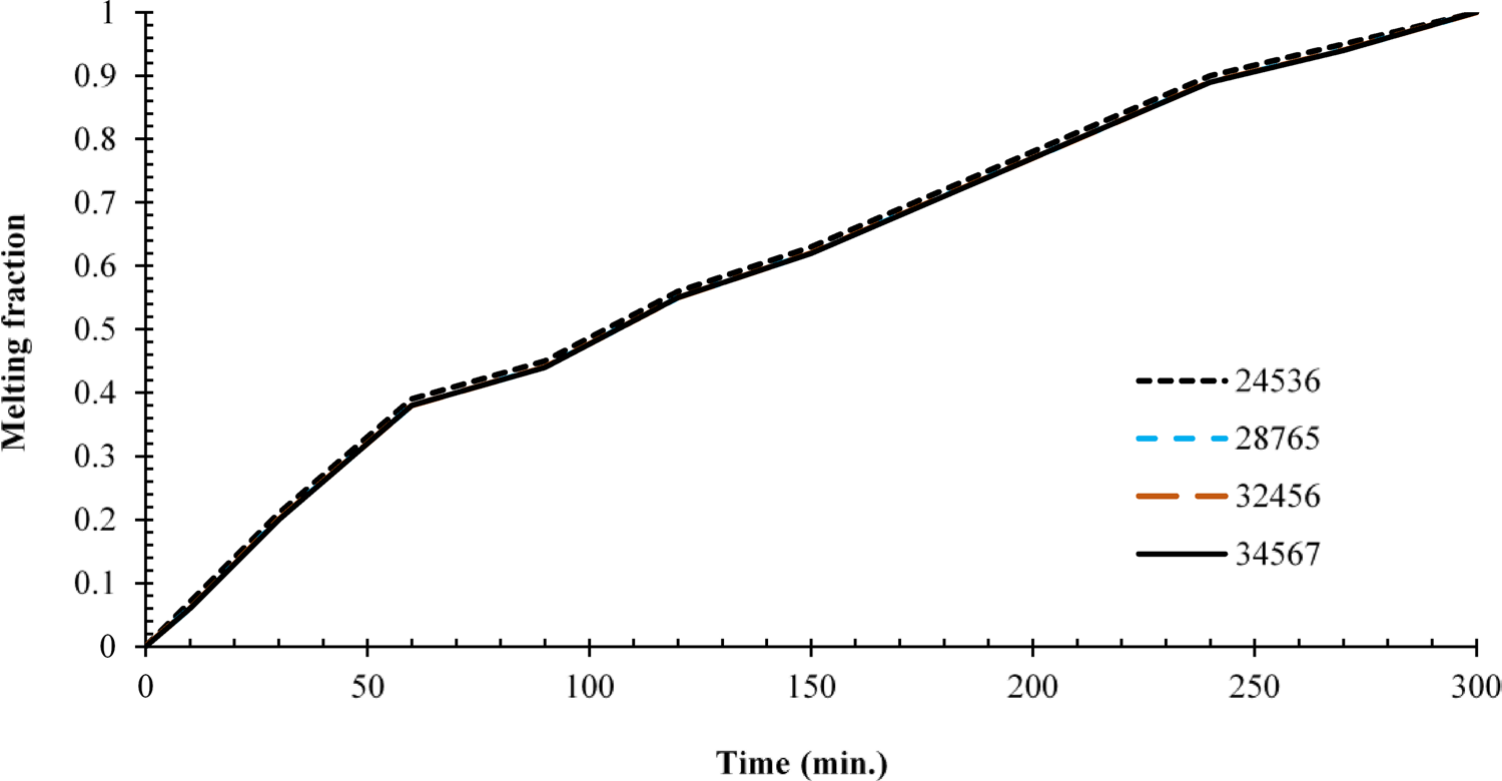
Grid independence without fins.

To evaluate the influence of mesh density on phase change behaviour within the specified geometric configuration, special attention was given to mesh independence, a critical preliminary step in computational fluid dynamics (CFD) studies. The primary objective was to accurately capture the evolution of the phase change process. Four distinct mesh densities were selected, corresponding to element counts of 24,536; 28,765; 32,456; and 34,567 and comparing the results of their phase change evolution. Following a detailed analysis of the results obtained from each mesh, it was found that the phase change evolution showed consistent behaviour across all cases. In other words, the simulations showed no deviations in the behaviour of the results among the various mesh arrangements, which indicated grid independence. This consistency indicates that the solution is grid-independent, meaning that variations in mesh density did not significantly affect the computational results. For subsequent simulations, the mesh with 28,765 element mesh was selected to run further simulations because it maximised performance since it was computationally efficient and highly accurate to be used to produce valid enough results without any waste of computational resources. Thus, this configuration offered an optimal balance between computational efficiency and solution accuracy, enabling faster processing without compromising result accuracy. The mesh configuration and independence analysis are illustrated in Fig. 3.

**Fig. 4:**
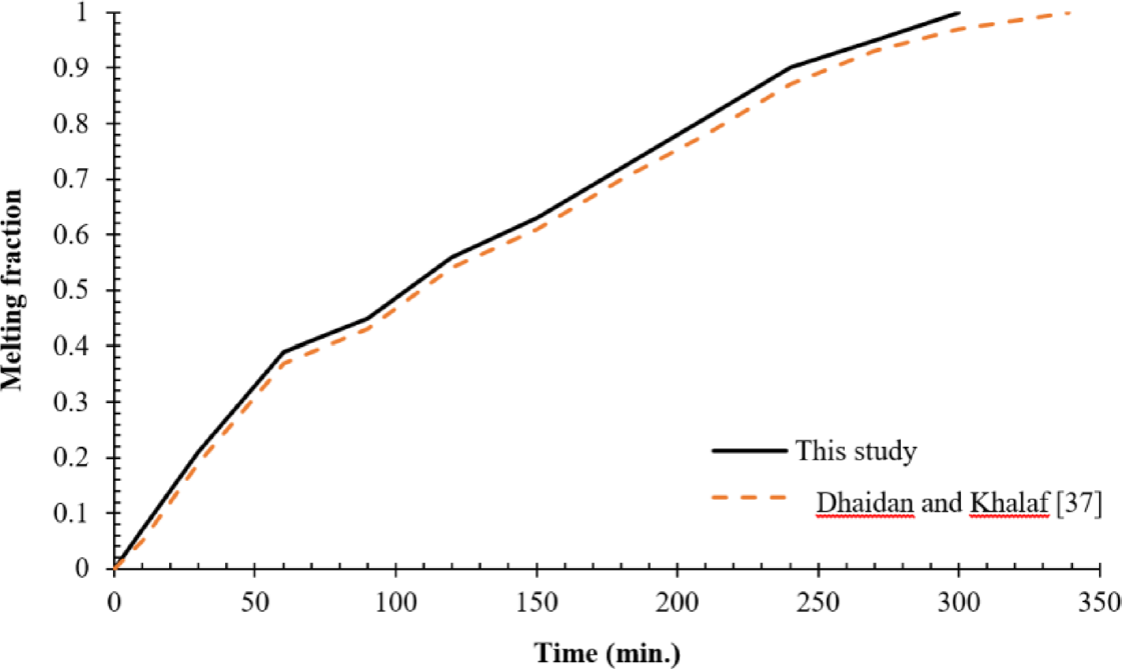
Comparison of the melting fraction versus operating time b\etween the present study and the numerical results reported by Dhaidan and Khalaf ^37^.

A CFD validation was performed by comparing the numerical results obtained from ANSYS/FLUENT 16 simulations with existed experimental data and theoretical predictions concerning the melting behaviour of PCMs. The validation focused on assessing the accuracy of the current numerical model by comparing its output with previously published experimental and numerical studies. In particular, the present study was benchmarked against a numerical investigation conducted by Dhaidan and Khalaf^37^, which examined the phase transition in a cylindrical system. The stringent verification procedure is in concurrence with the generally accepted best practice concerning CFD and is additionally promoted by the fact that the current computed solutions agree to a high degree with experimentally measure benchmark values presented by Dhaidan and Khalaf^37^, which testifies to the validity of the current approach. The comparison focused on PCM temperature as a key parameter to evaluate the agreement between the simulated results and expected behaviour based on experimental and theoretical insights. The current boundary conditions of the current simulation comply with the literature on PCM (e.g., Dhaidan and Khalaf^37^, therefore, are validated by the experimental observations (Fig. 4), and assured the reliability of our findings. As shown in Fig. 4, when water was circulated through the cylinder, a strong correlation was observed between the present results and those reported in the referenced study. This agreement confirms the accuracy and reliability of the current numerical approach. The consistency between both sets of results highlights the effectiveness of the simulation methodology employed in this research. Furthermore, the outcomes of the present research closely match the benchmark data, reinforcing the validity of the proposed modelling framework.

Referring to the enthalpy-porosity approach, it was adopted in ANSYS/FLUENT 16 through a source term coupling energy and momentum equation fields (Eq. 8) while using a constant mushy zone (C = 105). Grid independence tests were thoroughly performed to validate the accuracy of the model (Fig. 3) and the model was also benchmarked against experimental and numerical results (Fig. 4) that agreed well with each other’s in the evolution of melting fraction. Such a method has been widely used in PCM literature and the consistency of the current findings with published results is encouraging with regards to its validity in descriptions of phase change behaviour, such as conduction–convection transitions and non-linear interface phenomena.

## Results and discussion

This study investigates four scenarios involving a cylindrical cell filled with PCM. To evaluate the influence on the melting behaviour and the time required for complete melting, the first case considers the cell without a copper rod, while the remaining cases include variations with copper rod integration. In this aspect, it should be noted that the current simulation program considered clearly natural convection via buoyancy forces when melting the material. Enthalpy-porosity model in ANSYS/FLUENT 16 introduced the Boussinesq approximation to trace the density changes with resulting temperature gradients, of prime importance to the construction of natural convection.

### Case one (cell without rods)

**Fig. 5:**
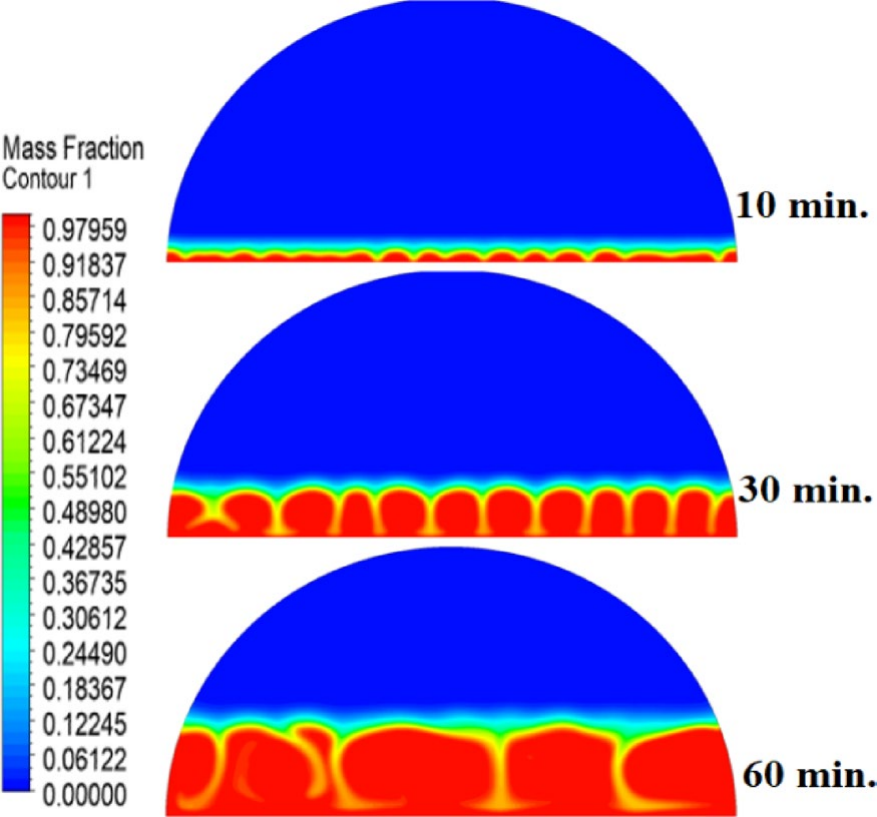
Predicted evolution of the melting process in the absence of copper rods, showing conduction-dominated heat transfer near the wall and slower melting in the central region.

**Fig. 6:**
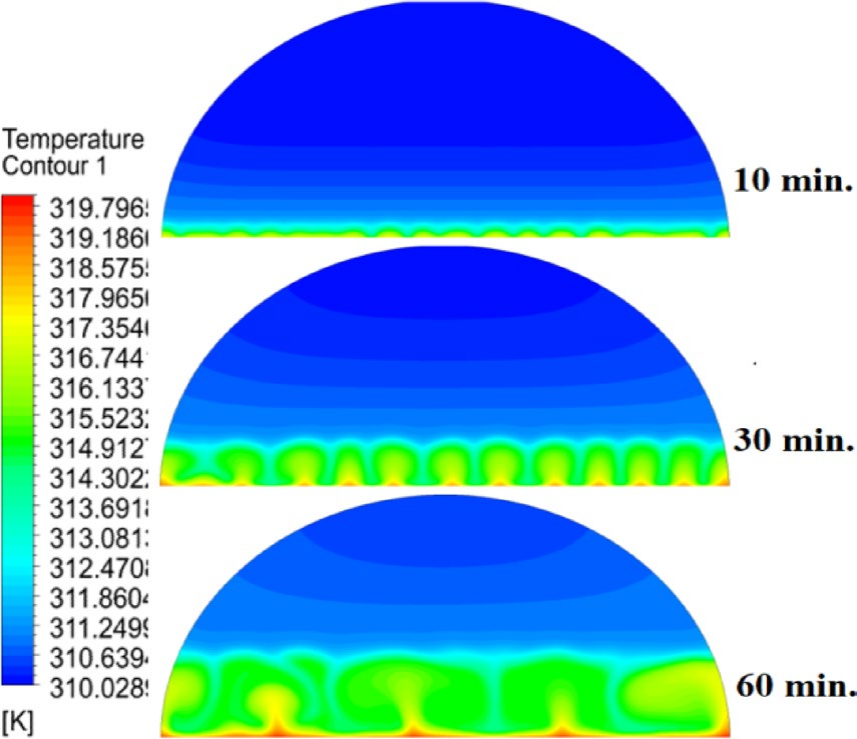
Temperature distribution across the cell (without rods), highlighting steep thermal gradients near the heated wall and reduced heat diffusion toward the centre.

**Fig. 7:**
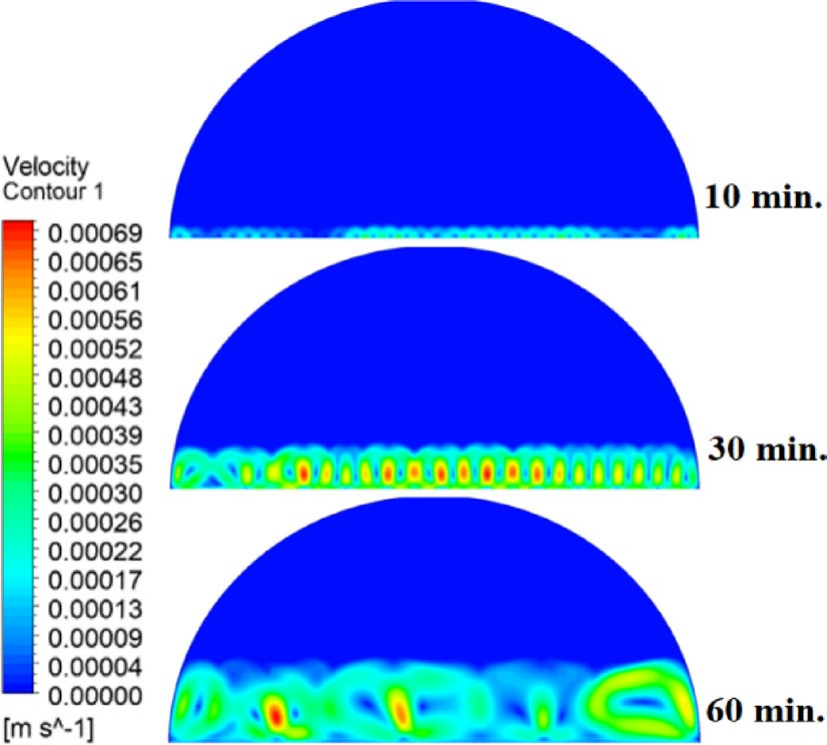
Velocity field during melting (without rods), illustrating strong convection currents near the wall and stagnant flow in the core, limiting heat transfer efficiency.

In this case, the cylindrical cell was analysed without copper rods to establish a baseline for the melting behaviour of the PCM, paraffin wax (RT42). Figure 5 illustrates the progression of the melting process, where heat transfer initiates near the heated wall via conduction and gradually extends into the interior of the PCM. As the distance from the wall increases, the melting rate significantly decreases, indicating a transition from conduction-dominated to convection-dominated heat transfer. This behaviour highlights the limitations of natural convection in distributing heat efficiently throughout the PCM, leading to uneven melting and extended completion times. Figure 6 presents the temperature distribution within the cell, revealing a steep thermal gradient adjacent to the heated wall, which progressively flattens toward the centre. The high-temperature region remains localized near the wall, while the cooler central zone reflects the inefficiency of heat diffusion in the absence of conductive enhancement. This temperature profile underscores the dominant role of natural convection, which is less effective at achieving uniform thermal distribution compared to conduction-enhanced mechanisms. The reduced heat propagation explains the prolonged time required for complete melting in the no-rod configuration. The velocity distribution shown in Fig. 7 further clarifies the fluid dynamics during the melting process. Fluid motion is most prominent near the heated wall, where buoyancy-driven convection currents are strongest. However, these currents diminish toward the centre, resulting in stagnant regions that hinder heat transfer. The observed velocity vectors align with the temperature gradients, confirming that natural convection alone is insufficient to ensure rapid or uniform melting. Collectively, Figs. 5, 6 and 7 demonstrate the inefficiency of the configuration without copper rods, emphasizing the necessity of conductive enhancements to optimize thermal performance and accelerate the melting process.

### Case two (with rods 10 mm)

**Fig. 8:**
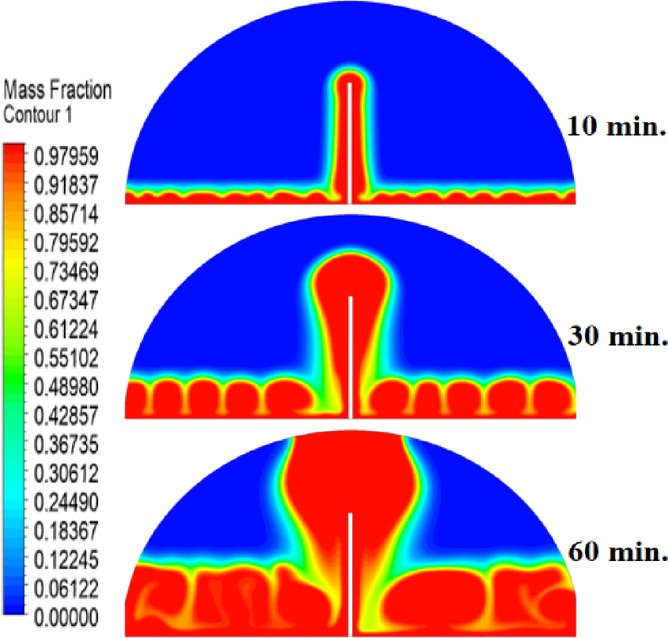
Melting evolution with a 10 mm rod showing enhanced conduction.

**Fig. 9:**
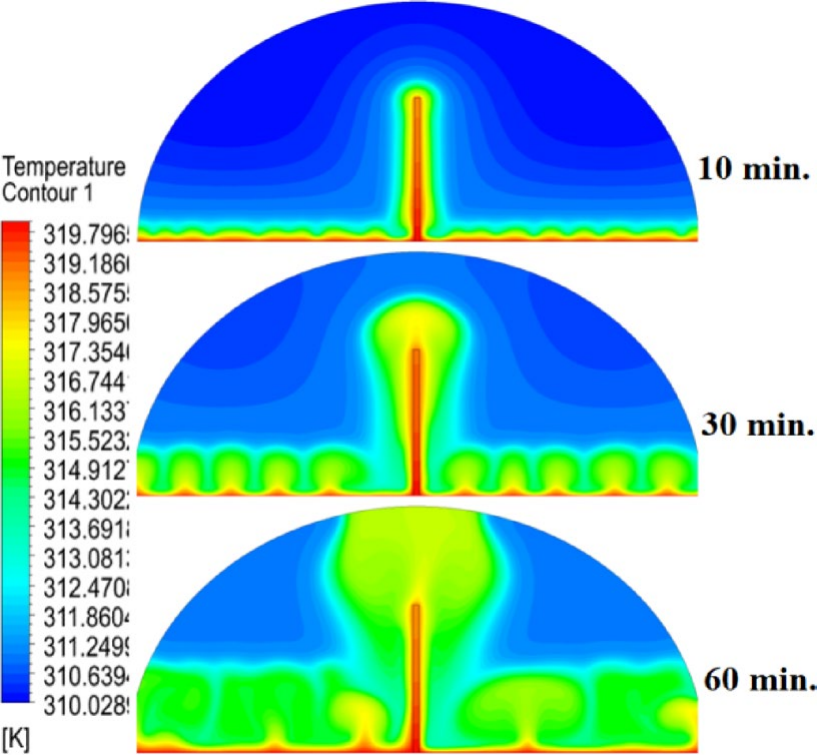
Temperature distribution with reduced gradients near the rod.

**Fig. 10:**
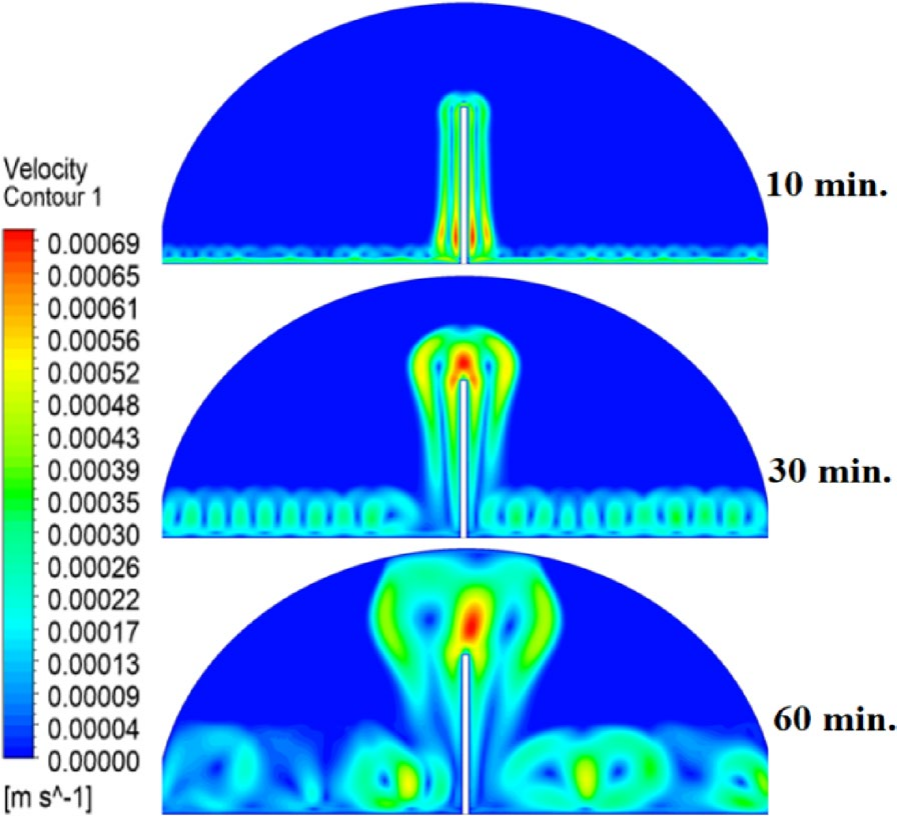
Velocity field revealing intensified convection around the rod.

The introduction of a 10 mm copper rod significantly altered the melting dynamics of the PCM, as illustrated in Figs. 8, 9 and 10. Figure 8 shows that the copper rod enhanced heat conduction from the heated wall into the PCM, resulting in a more uniform melting front compared to Case 1 (no rod) configuration. Although natural convection remained active in regions farther from the rod, the conductive pathway provided by the copper rod reduced reliance on buoyancy-driven flow, thereby accelerating heat propagation. However, the limited length of the rod confined its influence to the immediate vicinity, leaving the outer PCM regions still dependent on slower convective heat transfer. The temperature distribution in Fig. 9 reveals a distinct thermal pattern, with the copper rod acting as a high-conductivity channel that reduced thermal gradients in its surrounding area. The rod facilitated more rapid heat diffusion into the PCM, but its influence diminished with distance, resulting in a thermal plateau in regions beyond the rod’s effective reach. This behaviour confirms that, while the 10 mm rod improved local heat transfer, it did not uniformly accelerate melting across the entire cell. The persistence of cooler zones in the upper and outer regions suggests that longer or multiple rods may be required to achieve more comprehensive thermal enhancement. The velocity field in Fig. 10 further clarifies the effect of the copper rod on the melting process. Convective currents intensified around the rod, where heat was rapidly transferred to the surrounding PCM, generating localized vortices that enhanced mixing and reduced thermal stratification. However, the overall fluid motion remained weak in areas distant from the rod, consistent with the limitations observed in the temperature field. The combined analysis of Figs. 8, 9 and 10 indicates that the 10 mm rod improved melting efficiency by approximately 25%. Nevertheless, its localized impact underscores the need for optimization of rod dimensions or configurations to achieve more uniform and accelerated phase change throughout the entire PCM domain.

###  Case three (with rods 15 mm)

**Fig. 11:**
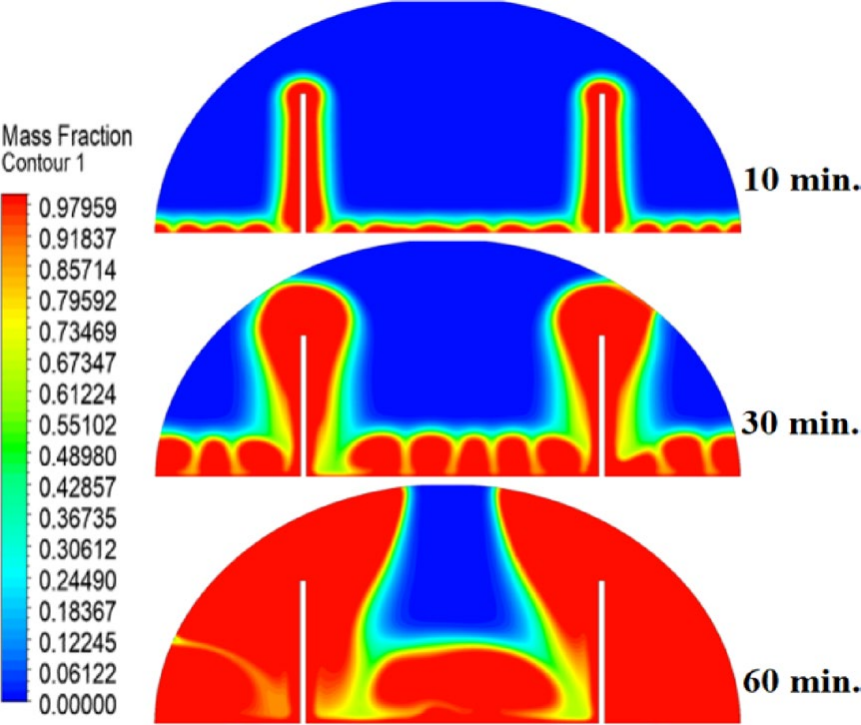
Melting progression with a 15 mm rod, showing deeper heat penetration.

**Fig. 12:**
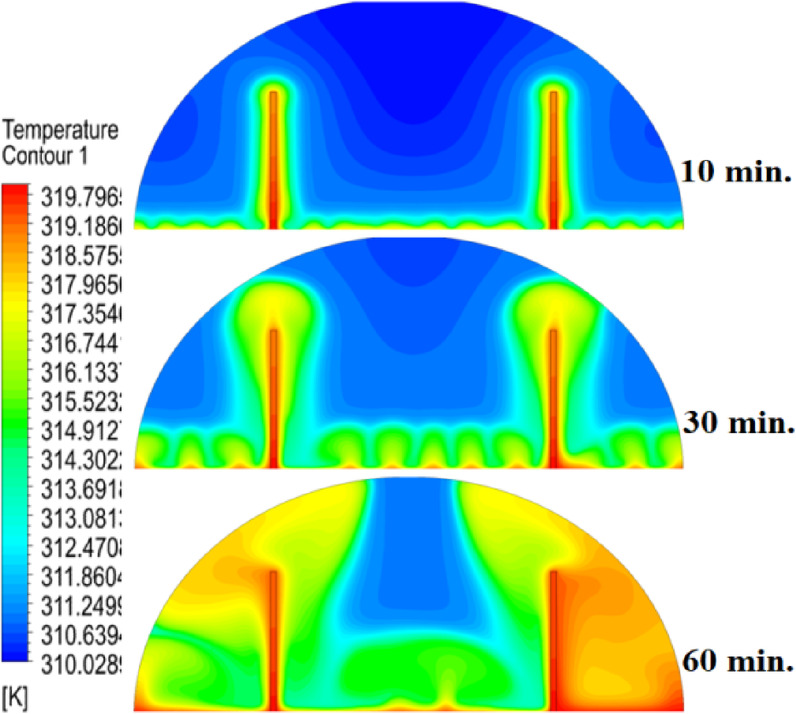
Smoother temperature distribution due to extended conductive pathways.

**Fig. 13:**
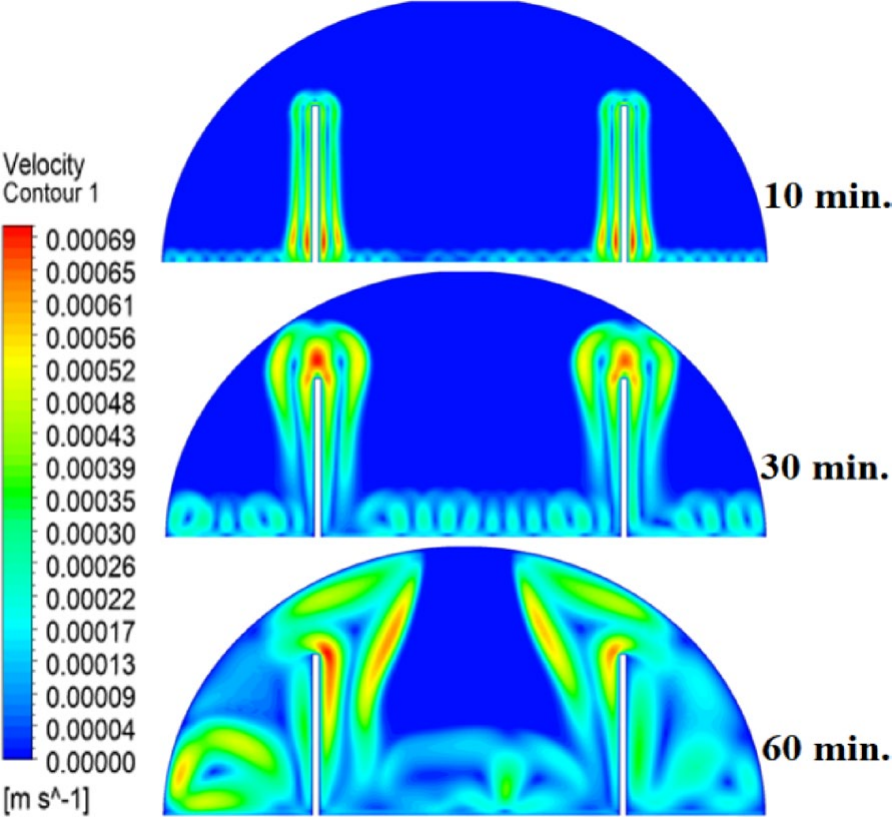
Enhanced velocity field with stronger convection near the rod.

The inclusion of a 15 mm copper rod further improved the melting performance of the PCM, as illustrated in Figs. 11, 12, and 13. Figure 11 shows a more advanced melting front compared to the 10 mm rod case, with the increased rod length enabling deeper heat penetration into the PCM. The conductive pathway established by the rod significantly diminished the dominance of natural convection, particularly in the lower and central regions of the cell. However, the upper portions of the PCM continued to exhibit slower melting, indicating that while the 15 mm rod enhanced overall efficiency, it did not completely overcome the limitations imposed by convection. The temperature distribution in Fig. 12 demonstrates a more uniform thermal profile, with the rod effectively bridging the gap between the heated wall and the PCM core. The extended length allowed heat to propagate further into the material, reducing thermal gradients and minimizing stagnant thermal zones. Despite this improvement, a temperature difference between the regions adjacent to the rod and those farther away, suggesting that although the 15 mm rod outperformed its shorter counterpart, full thermal uniformity would require even greater conductive enhancement. The velocity field in Fig. 13 reveals stronger convective currents in the vicinity of the rod, driven by enhanced heat diffusion. The flow patterns indicate improved mixing relative to the 10 mm rod case, particularly in the mid-regions of the cell. However, weaker currents persisted in the upper areas, underscoring the continued influence of natural convection in those zones. Figures 11, 12, and 13 confirm that the 15 mm rod reduced the total melting time by approximately 40%. This configuration achieved an effective balance between conduction and convection, while also highlighting the potential for further optimization using longer or multiple copper rods.

### Case three (with rods 20 mm)

**Fig. 14:**
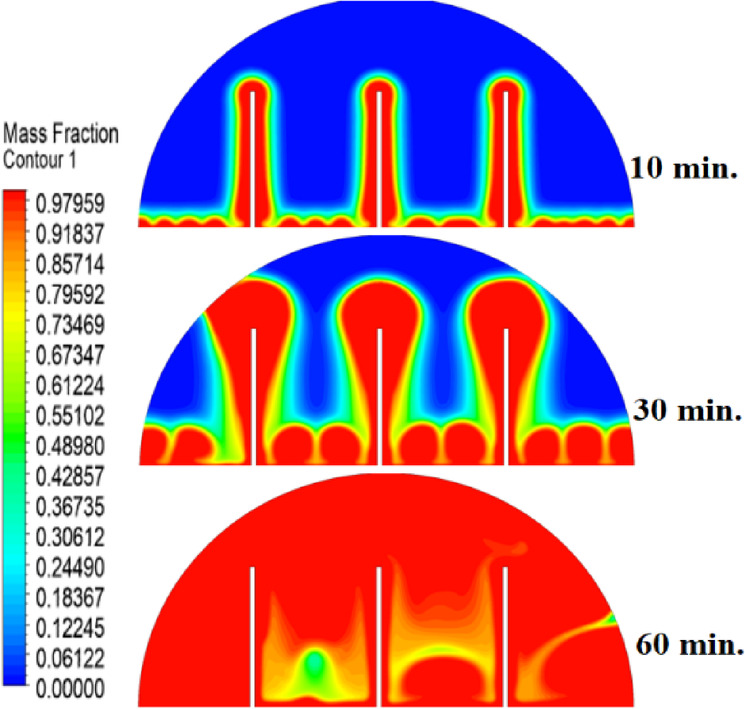
Complete melting front advancement with optimal 20 mm rod configuration.

**Fig. 15:**
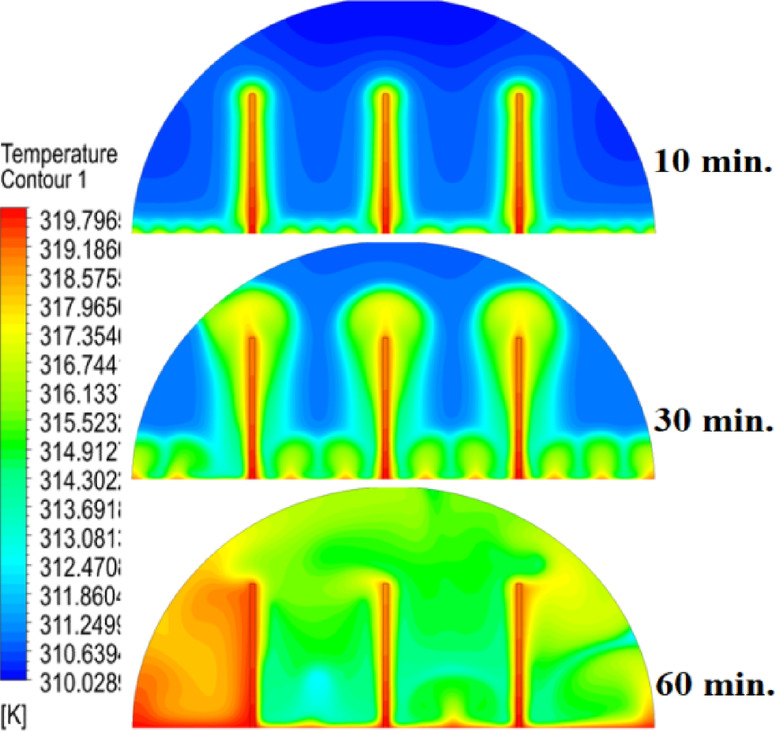
Nearly uniform temperature distribution showing minimal thermal gradients.

**Fig. 16:**
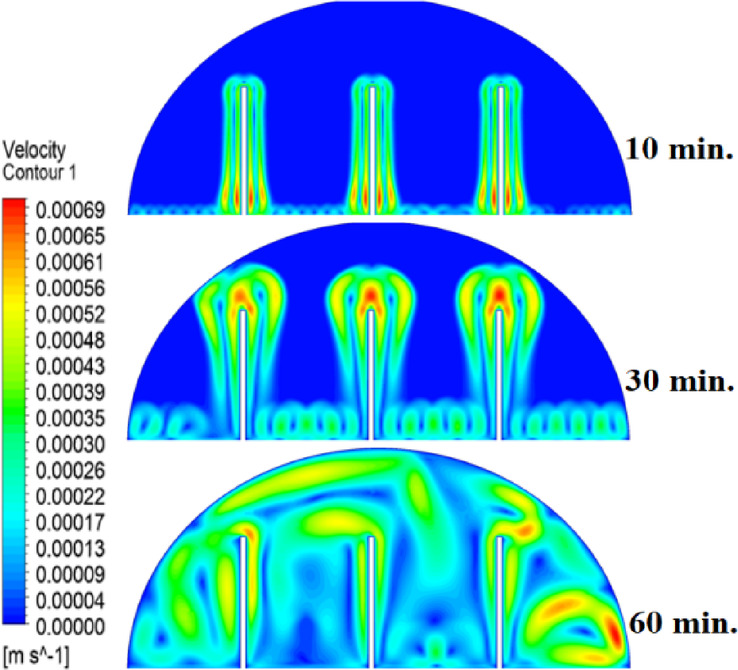
Balanced velocity field demonstrating conduction–convection synergy.

The 20 mm copper rod demonstrated the most significant improvement in melting efficiency among all tested configurations, as illustrated in Figs. 14, 15 and 16. Figure 14 shows a significantly enhanced melting front, with the extended rod length enabling near-complete heat penetration throughout the PCM volume. Conductive heat transfer dominates, as the rod effectively spans the entire cross-section of the cell, substantially reducing reliance on slower natural convection mechanisms. This configuration reduced the total melting time by 50% compared to Case 1, representing the optimal balance between conductive and convective heat transfer achieved in this study. The temperature distribution in Fig. 15 reveals the most uniform thermal profile observed across all cases. The 20 mm rod establishes an efficient thermal bridge that nearly eliminates temperature gradients in the central region, while maintaining only minimal differentials in the outermost areas of the PCM. This near-uniform heating marks a substantial improvement over the shorter rod configurations, although slight temperature variations persist near the top of the cell, where convective effects remain dominant. These thermal patterns suggest that a 20 mm rod approaches the practical limit for heat transfer enhancement within this specific geometric configuration. The velocity field in Fig. 16 exhibits well-organized convection patterns that complement the rod’s conductive performance. While convective currents are still present, their intensity and spatial distribution are more uniform than in the shorter rod cases. The overall reduction in velocity magnitudes indicates that conduction through the rod has largely supplanted the need for vigorous natural convection. This synergistic interaction between conduction and convection in the 20 mm rod case demonstrates how well-designed conductive elements can optimize phase change processes, achieving both rapid and uniform melting in thermal energy storage systems.

Referring to the above Figs. 6, 9, 12 and 15, which focused on demonstrating an in-depth spatial study regarding temperature distributions within the PCM, it can be stated that copper rods have significantly decreased temperature non-uniformity as they provided a conductive route where 20 mm rods displayed nearly flat thermal patterns (Fig. 15) and the non-rod case showed steep gradients (Fig. 6). This is guaranteed by the results of velocity fields (Figs. 7, 10, 13, 16), explicitly correlate rod length and improved heat diffusion, and finally concluded that conductive elements are used to alleviate PCMs dependency on the inefficiencies of natural convection.

To summarise the above findings, the current findings (Figs. 5, 6, 7, 10, 13, and 16) can ascertain that the mechanisms are very graphic, as the conduction and convection interact with each other, and the velocity fields depict circulation patterns created by buoyancy. It is worth noting that the 20 mm rod case has shown that with proper conductive enhancement, the natural convection dependency could be minimised, whereas the shorter rods still maintained a high convection influence. Such a two-stage mechanism is supported by the corresponding literature ^14,18,22^ and was tested against a set of experimental standards^37^, testifying to the validity of our approach. Furthermore, as the melting time can ascertain the duration required to achieve a complete melting (β = 1) of the PCM, intermediate liquid fractions, such as the 70% threshold (β = 0.7) were tested to address transient performance. These demonstrated that the 20 mm rod reached this milestone four times faster than the no-rod case (30 vs. 120 min).

## Comparison of three cases

**Fig. 17:**
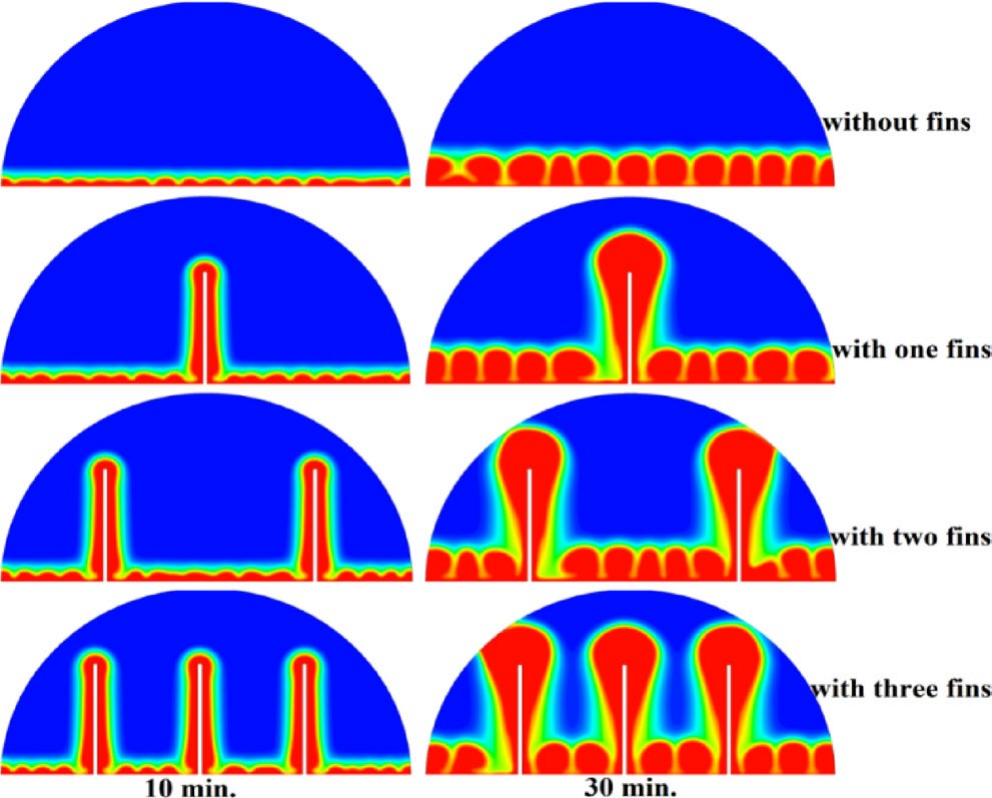
Progressive melting time reduction with increasing rod length.

**Fig. 18:**
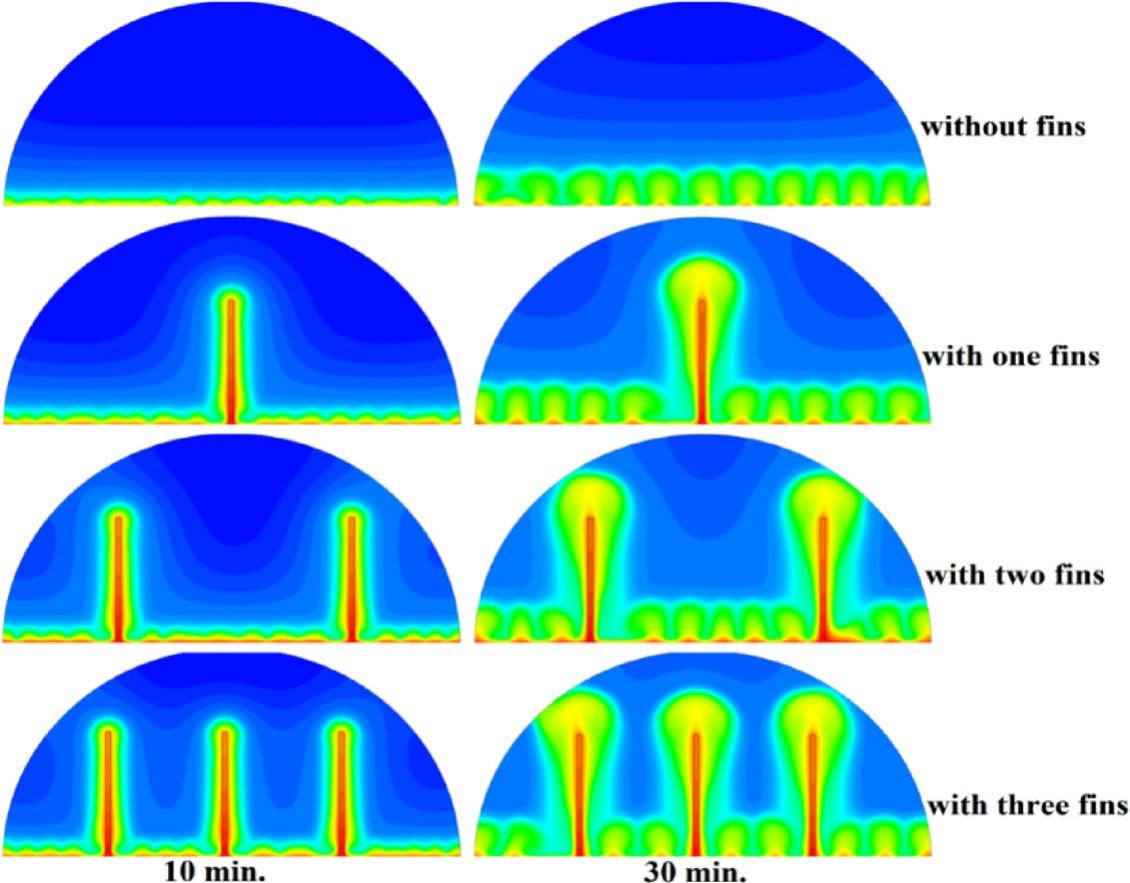
Thermal gradient improvement across different rod configurations.

**Fig. 19:**
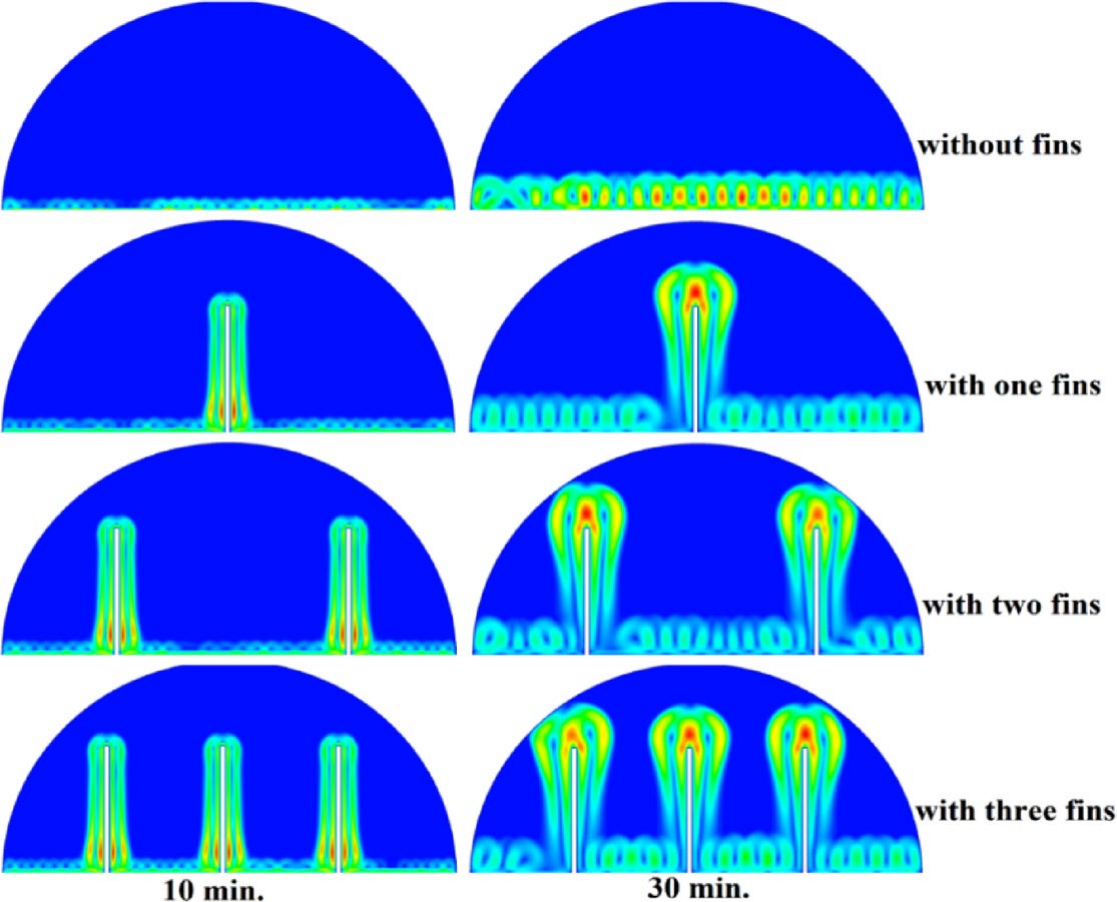
Comparison of the velocity between all cases, convection intensity inversely related to conductive enhancement.

The comparative analysis of all configurations, as illustrated in Figs. 17, 18 and 19, provides significant insights into the impact of copper rod integration on PCM melting dynamics. Figure 17 shows a clear progression in melting completion times: the non-rod case required 40 min, while the configurations with 10 mm, 15 mm, and 20 mm rods achieved complete melting in 30, 25, and 20 min, respectively. This trend highlights a direct correlation between rod length and thermal performance, with the 20 mm rod delivering the most substantial improvement, reducing melting time by 50% compared to the baseline case. Temperature distribution comparisons in Fig. 18 offer compelling evidence of the thermal bridging effect provided by the copper rods. The temperature profiles become increasingly uniform with longer rods, with the 20 mm rod case exhibiting the flattest thermal gradient. This demonstrates how extended conductive pathways can effectively reduce the thermal resistance inherent in PCMs. Notably, the 20 mm rod nearly eliminates the steep temperature drop observed in the no-rod configuration. However, the improvement in thermal performance follows a nonlinear trend, suggesting diminishing returns beyond a certain optimal rod length. The velocity field comparisons in Fig. 19 reveal an interesting contrast in heat transfer mechanisms. Natural convection currents are most vigorous in the no-rod case but decrease in intensity as rod length increases. In the 20 mm configuration, the flow is weaker yet more organized. This inverse relationship between convection strength and overall melting efficiency underscores the superiority of conduction-enhanced heat transfer in PCM systems. Collectively, the data suggest that while natural convection contributes to heat distribution, it is insufficient for achieving rapid and uniform melting. Strategic conductive enhancement through copper rods offers a more effective and reliable approach to thermal management in phase change systems.

**Fig. 20:**
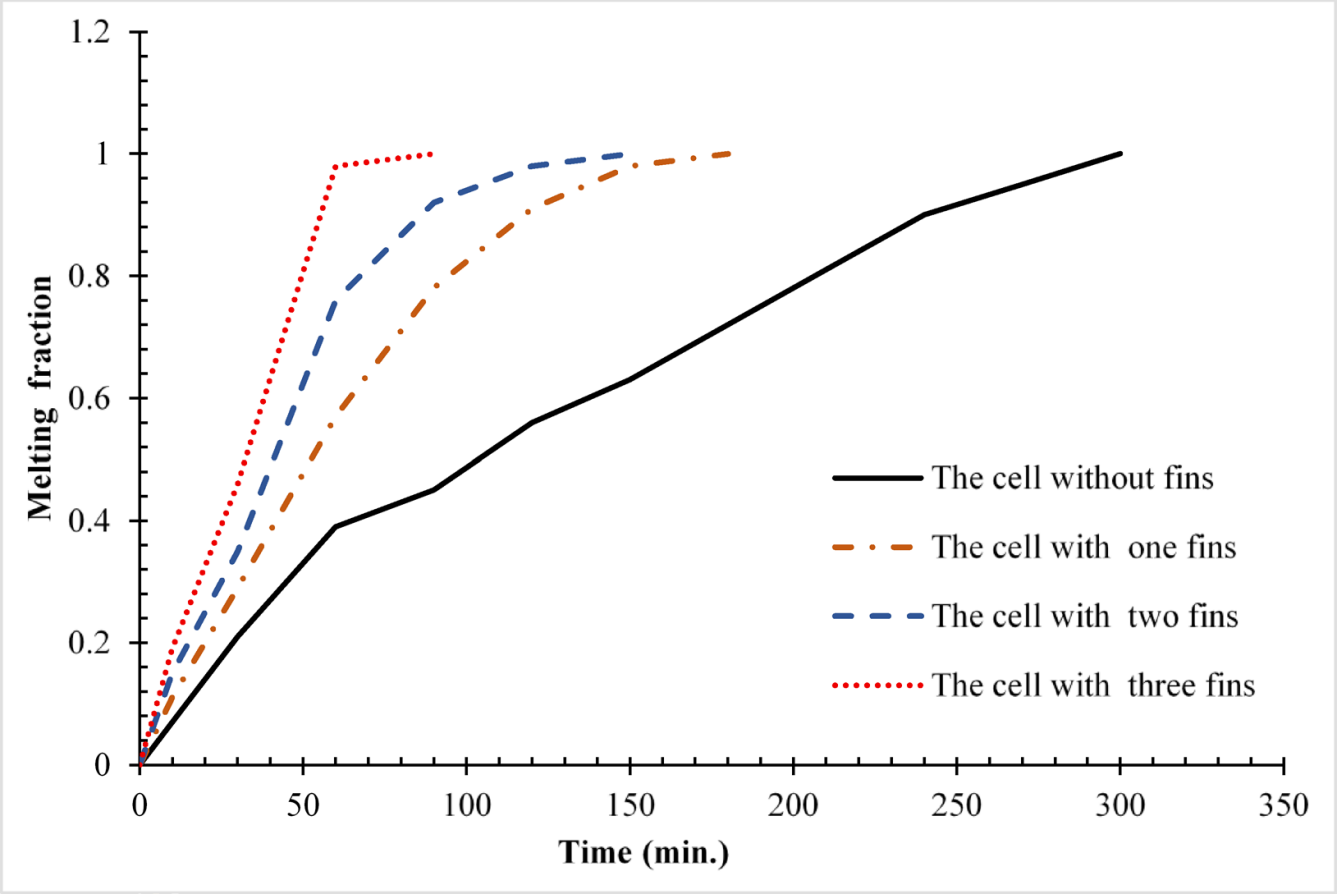
Variation of liquid fraction between all cases.

Figure 20 presents a detailed quantification of melting performance through the temporal evolution of the liquid fraction across all test configurations. The baseline case (no rod) exhibited the slowest phase transition, requiring 300 min to achieve complete melting (β = 1). In stark contrast, configurations incorporating copper rods demonstrated substantial thermal performance enhancements: the 10 mm, 15 mm, and 20 mm rod cases achieved full melting in 180, 150, and 90 min, respectively, corresponding to time reductions of 40%, 50%, and 70%. Particularly noteworthy is the performance of the 20 mm rod configuration, which reached a 70% liquid fraction (β = 0.7) in just 30 min—four times faster than the 120 min required by the baseline case to reach the same phase transition milestone. These results reveal important nonlinearities in the thermal enhancement mechanism. While the initial 10 mm rod reduced total melting time by 40% (a 120-min improvement), subsequent 5 mm increments yielded diminishing returns: the 15 mm rod provided an additional 10% improvement (50% total reduction), and the 20 mm rod a further 20% gain (70% total). This nonlinear scaling suggests the existence of an optimal rod length between 15 and 20 mm, beyond which the marginal thermal benefits may not justify the additional material and manufacturing costs. The performance metrics underscore that the most pronounced per-millimetre gains occur within the 0–15 mm range, with the transition from 10 to 15 mm offering the highest cost–benefit efficiency compared to further extensions. These findings provide valuable insights for the optimization of thermal energy storage system design, particularly in balancing performance gains with material efficiency.

To quantify the nonlinear scaling behaviour, the interaction effect between the length of the rod and the reduction of the melting time was measured by comparing the change of the data in percentage: the 10 mm rod resulted in a reduction of 40% (120 min difference), the 15 mm rod added even more, 10% (30 min difference), and the 20 mm rod provided 20% (60 min difference) more as well. This diminishing output was clearly displayed in Fig. 20 (liquid fraction vs. time) where a flat out line logarithmic-like trend was shown after a certain point although the performance even after 15 mm was showed to at least remain unchanged. Although no specific correlation was tested, because the measured lengths were discrete, the data points highly indicate that the scaling follows a power-law or exponential decay model relationship because the shorter the length the more disproportionate is the gain. This experimental finding can be said to be consistent with theoretically expected saturation of conductive heat transfer and whereby, it brings us practical hints that can be employed in the practical design of rods.

Referring to the current numerical simulations, ANSYS/FLUENT 16 enthalpy porosity model was used under close care of the mushy zone constant (C = 105) to allow stability and convergence on every rod tested including the largest tested aspect ratio (20 mm rod). This approach was found sound as assessed in the grid independence study (28,765 elements), as well as during comparison against the experimental data at Dhaidan and Khalaf^37^. Although some smaller iterative correction was necessary to undo the localized nonlinearities that occur at the solid–liquid interface, there were no overriding instabilities, witnessed through the steadily reducing residuals (and scored < 10^–6^ in energy and < 10^–5^ in continuity and momentum) and the reliability of the associated results compared to the physical common sense. In the liquid fraction curves (Fig. 20), the fact that there is no evidence of divergence or the oscillatory nature of the liquid fraction further added to the rigidity of the utilised methodology.

## Conclusions

This study investigated the enhancement of PCM melting performance through the integration of copper rods within a horizontally oriented hemispherical cell. A novel numerical approach was employed, combining enthalpy–porosity modelling with ANSYS/FLUENT 16 simulations to quantify the impact of conductive enhancements on heat transfer dynamics. Key findings include:

- The 10 mm, 15 mm, and 20 mm copper rods reduced total melting time by 40% (from 300 to 180 min), 50% (to 150 min), and 70% (to 90 min), respectively, compared to the no-rod baseline (300 min).
- While the 10 mm rod achieved a 120-min reduction in melting time, subsequent 5 mm increases yielded diminishing returns. While, the 15 mm rod contributed an additional 30 min of improvement, and the 20 mm rod an additional 60 min, suggesting an optimal rod length in the 15–20 mm range.
- The 20 mm rod shifted the dominant heat transfer mechanism from convection to conduction, significantly reducing reliance on slower, buoyancy-driven flows by over 50%.
- A 70% liquid fraction (β = 0.7) was reached in just 30 min with the 20 mm rod, compared to 120 min in the no-rod case, demonstrating near-complete thermal bridging across the PCM volume.
- The 15 mm rod achieved 85% of the maximum performance gain (50% reduction in melting time) while using 25% less material than the 20 mm rod, indicating strong potential for practical, cost-efficient deployment.

These results underscored the transformative role of conductive enhancements in PCM-based thermal energy storage systems. Copper rods function as thermal “highways”, effectively bypassing the inherent limitations of natural convection. The observed nonlinear relationship between rod length and melting efficiency provides critical guidance for system design, emphasizing that material optimization, not mere maximisation, is essential for cost-effective thermal management. Future research should include experimental validation of these findings, exploration of hybrid enhancement strategies (e.g., combining rods with nanoparticles), and investigation into more economical alternatives to copper. Additionally, scaling analyses for industrial applications and long-term thermal cycling tests are necessary to assess practical feasibility and durability.

## Data Availability

The data supporting the findings of this study can be accessed from corresponding author upon reasonable request.
